# Myocardial and blood T1 quantification in normal volunteers at 3T

**DOI:** 10.1186/1532-429X-13-S1-P51

**Published:** 2011-02-02

**Authors:** Christopher T Sibley, Justin Huang, Martin Ugander, Abiola Oki, Jing Han, Marcelo S Nacif, Andreas Greiser, Daniel R Messroghli, Peter Kellman, Andrew E Arai, David A Bluemke, Songtao Liu

**Affiliations:** 1 NIH Clinical Center, Bethesda, MD, USA; 2 NHLBI, Bethesda, MD, USA; 3 U. S. FDA, Rockville, MD, USA; 4 Siemens AG, Erlangen, Germany; 5 Franz-Volhard-Klinik Charité, Berlin, Germany

## Introduction

T1 mapping is a novel quantitative method for myocardial tissue characterization. Reference values of myocardium and blood have been established at 1.5T (Messroghli, MRM, 2007). CMR at 3T is a rapidly maturing field, and T1 values generally increase with magnetic field strength, making it necessary to establish new reference values at this higher field strength. The aim of this study is to establish pre- and post-contrast myocardium and blood reference T1 values at 3T.

## Methods

Normal volunteers (n = 9, 7 male, age 39±12 years) without cardiovascular disease were scanned at 3T (Verio, Siemens) with a 32-channel coil. GRE shimming was used to improve B1 inhomogeneity and transmitter frequency was adjusted according to frequency scout. Modified Look-Locker inversion recovery (MOLLI) images with 2 inversion-recovery blocks were acquired at mid-ventricular short axis view. Eight images were acquired in eleven heart beats with the following parameters: spatial resolution = 1.75×1.75×8 mm on a 256×180 matrix, TI initial = 110ms, TI increment = 80ms, flip angle = 35°, TR/TE=2.4/1.05ms. All subjects were administered Magnevist (0.15mmol/kg), and multiple post-contrast MOLLI scans were performed at the same pre-contrast position from 3.5-23.5 minutes after contrast injection. T1 maps were generated using MRMap (Messroghli, BMC Med Imaging, 2010). Average blood pool and myocardial T1 values were measured using QMass (Medis), and results were corrected for heart rate.

## Results

**Figure 1:**
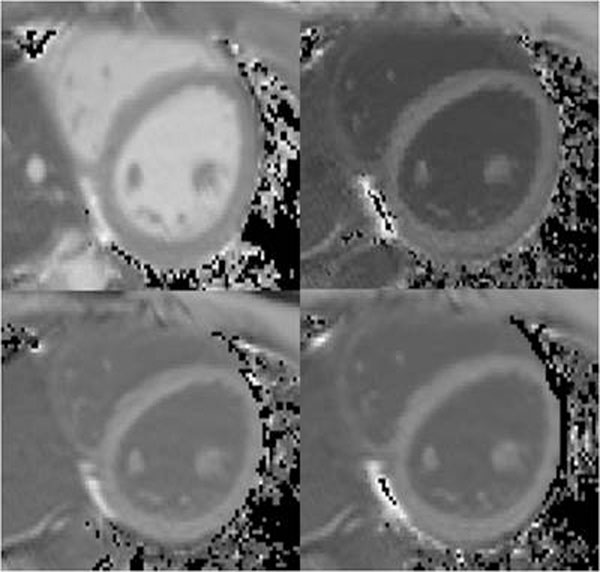
Mid-ventricular short axis T1 maps. Clockwise from top left: pre-contrast, 3.5 minutes post-contrast, 13.5 minutes post-contrast, 23.5 minutes post contrast.

**Figure 2:**
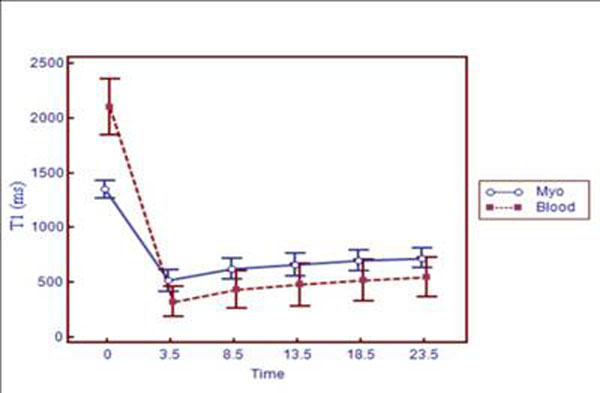
Recovery of absolute myocardial and blood T1 (mean±sd) from 0 min (pre-contrast) to 23.5min after 0.15mmol/kg of Gd-DTPA.

All Pre- and post-contrast images were interpretable for T1 mapping and contained few artifacts (Figure 1). Mean pre- and post- contrast myocardial and blood T1 value was compiled in Figure 2. Mean pre-contrast myocardial T1 was 1347±37 ms, and blood T1 was 2076±125 ms. Myocardial T1 values at 3T were 43% greater than previously reported at 1.5T and consistent with results using different methods (Stanisz, et al, MRM, 2005, Sharma, et al, JMRI, 2006).

## Conclusions

High resolution T1 mapping using MOLLI is feasible at 3T, consistently yielding images of adequate quality for measurement. Pre- and post-contrast normal myocardial and blood T1 values are markedly higher at 3T than at 1.5T. T1 recovery in both myocardium and blood is in near-equilibrium between 3.5 minutes and 23.5 minutes post contrast.

